# Proton Pump Inhibitors and Oral–Gut Microbiota: From Mechanism to Clinical Significance

**DOI:** 10.3390/biomedicines12102271

**Published:** 2024-10-07

**Authors:** Xian Zhang, Qing Li, Siyuan Xia, Yan He, Yuqiang Liu, Jinlin Yang, Xue Xiao

**Affiliations:** 1 Department of Pathology, Sichuan University-University of Oxford Huaxi Joint Centre for Gastrointestinal Cancer, Frontiers Science Center for Disease-Related Molecular Network, West China Hospital, Sichuan University, Chengdu 610041, China; zhangxian@wchscu.cn; 2 Department of Gastroenterology and Hepatology, Sichuan University-University of Oxford Huaxi Joint Centre for Gastrointestinal Cancer, Frontiers Science Center for Disease-Related Molecular Network, West China Hospital, Sichuan University, Chengdu 610041, China; liqing@wchscu.cn (Q.L.); siyuanxia98@163.com (S.X.); litbit@163.com (Y.L.); mouse-577@163.com (J.Y.)

**Keywords:** proton pump inhibitors, oral microbiota, gut microbiota, oral–gut translocation, digestive diseases

## Abstract

Proton pump inhibitors (PPIs) are some of the most commonly prescribed drugs worldwide, but there are increasing concerns about digestive complications linked to PPIs. Next-generation sequencing studies have suggested that PPIs can significantly affect the composition of the gut microbiota, which in turn may substantially contribute to the development of these complications. Recently, emerging evidence has suggested that the translocation of oral microbes into the gut may be the primary mechanism underlying the alterations in the gut microbiota induced by PPIs in the presence of gastric acid suppression and impaired oral–gut barrier function. Moreover, the significance of oral–gut microbial translocation in health and disease conditions has gained increasing recognition. Consequently, it is imperative to enhance our understanding of the functions of the oral–gut microbiota axis in digestive disorders associated with PPI therapies. This review aims to summarize current research findings and further elucidate the contribution of the oral–gut microbiota to the pathogenesis of PPI-related digestive diseases. We aim to provide a theoretical foundation for future therapeutic and preventive strategies targeting PPI-related digestive complications through modulation of the oral–gut microbiota.

## 1. Introduction

Proton pump inhibitors (PPIs), firstly introduced in the late 1980s, have gained widespread clinical application for the management of upper gastrointestinal conditions characterized by excessive gastric acid secretion [1,2]. The acidic environment prompts the transformation of PPI prodrugs into active metabolites, which can irreversibly block the hydrogen–potassium (H^+^-K^+^) ATPase activity of parietal cells, thus preventing gastric acid secretion and increasing the pH, in turn promoting tissue repair [3]. Approximately one-quarter of the adult population is prescribed PPIs, with an estimated 25–70% of these prescriptions considered inappropriate [4]. Recent research has shed light on the potential health risks associated with the prolonged and excessive prescription of PPIs, leading to growing concerns about their overutilization [1,5,6]. In particular, PPIs have been linked to an elevated risk of various digestive diseases, including inflammatory bowel disease (IBD), gastrointestinal malignancies, and enteric infections such as *Clostridioides difficile* infection (CDI) [7,8,9,10]. Given the widespread prescription of these medications, these potential complications warrant further attention.

PPIs can affect the gut microbiota. Two large cohort studies of more than 1000 individuals revealed that the administration of PPIs disturbed the gut microbiota, with up to 20% of the identified taxa showing changes [11,12]. Disruption of the gut microbiota might increase the risk of disease or further exacerbate existing diseases [5,13]. Therefore, elucidating the specific alterations in the gut microbiome associated with PPI usage, as well as unraveling the underlying mechanism, could play a vital role in understanding the development of drug side effects.

The significance of oral–gut microbial translocation in relation to health and disease has been increasingly recognized in recent years. The oral cavity and gastrointestinal tract are the most important habitats for human microbiota. The oral cavity serves as the entryway to the gastrointestinal tract, and there was a previous belief that microbes residing in the oral cavity were eliminated by stomach acid and bile, preventing them from reaching the lower parts of the digestive tract [14,15]. Recent data indicate that the oral microbiota can translocate to and then colonize the intestine [16,17]. Ectopic colonization of oral microbes in the gut has been associated with various diseases, such as IBD, Alzheimer’s disease, and colorectal cancer (CRC) [18,19]. Our recent findings indicate that PPIs could facilitate the translocation of bacteria from the oral cavity to the intestines [17]. Therefore, it is expected that the association between PPI-induced alterations in the gut microbiota and their corresponding roles in different digestive diseases can be more rationally understood by integrating the concept and mechanism of the oral–gut microbial axis. This review aims to assess the role of the oral–gut microbiota in individuals taking PPI medications, and its significance for PPI-related digestive diseases. We also discuss changes in specific strains of microbes linked to certain diseases and the potential mechanisms by which these microbes may contribute to related digestive conditions.

## 2. Impact of PPIs on Oral and Gut Microbiota

### 2.1. PPIs and Oral Microbiota

The oral cavity is a primary gateway to the human body and harbors an incredibly rich and diverse microbial community, comprising mainly six phyla with over 700 distinct species [19]. Of those, Streptococcus, Prevotella, and Rothia are the predominant genera [20]. Currently, several studies have presented data about how taking PPI medications affects the microbial communities in the oral cavity (Table 1).

Tsuda et al. obtained saliva from 18 outpatients taking PPI therapies (PPI users) long-term for functional dyspepsia or gastroesophageal reflux disease (GERD) and 27 healthy individuals not taking PPIs (non-PPI users) for the microbiota analysis. Using the pyrosequencing method, it was found that PPIs did not significantly impact microbial species richness or microbial composition at the genus level. However, PPI users exhibited a more diverse range of microbial species present in their saliva when compared to non-PPI users [21]. Mishiro et al. prospectively examined the effect of PPIs on oral microbial communities in ten healthy volunteers. After a four-week administration of 20 mg esomeprazole, a significant reduction in the Shannon diversity index and significant differences in beta diversity between PPI users and non-users were observed in the microbiome from saliva samples. However, no such changes were observed in the periodontal pocket sample. Moreover, at the genus level, taxon-based analysis indicated that PPI administration decreased the abundance of Neisseria, Veillonella, and Haemophilus in the saliva, and increased the abundance of Leptotrichia and Fusobacterium in the periodontal pocket samples [22]. Kawar et al. examined the salivary microbiome in individuals with GERD who were chronically treated with or without PPIs, and compared it to that of healthy controls (defined as free of GERD and not using PPIs). The analysis revealed no significant variation in alpha diversity across the three cohorts, while notable differences were identified in the taxonomic profiles of the salivary microbiomes. After controlling for potential confounding factors such as age, periodontal status, and edentualism, all of which may influence the composition of salivary bacteria, 17 species and subgenera were found to exhibit significantly different abundances between the GERD patients not receiving PPIs and the healthy control group. Multivariate analysis further indicated that four of the 17 bacteria taxa, namely *Prevotella pallens*, *Prevotella melaninogenica*, Leptotrichia, and *Solobacterium moorei*, were reduced in the GERD patients. In contrast, the salivary taxa profiles of the subset of GERD patients chronically treated with PPIs were similar to those of the controls. Based on these findings, the researchers hypothesized that either GERD itself or the use of PPIs could be associated with alterations in the oral microbiome. When present together, the effects of PPIs on the oral microbiota seem to be minimized [23]. Rosen et al. obtained oropharyngeal fluid from 116 children with chronic coughs, and found that PPI administration within the past 24 h (59 children) did not alter microbial diversity, but increased the prevalence of Butyrivibrio and increased the abundance of 10 genera including Allobaculum, Bifidobacterium, Cloacibacterium, Janthinobacterium, Ralstonia, Rhodobacter, Rhodoferax, Streptococcus, Yersinia, and Zoogloea [24].

Although the results from current studies are not consistent, present evidence indicates that PPIs could alter the oral microbial composition.

**Table 1:** Summary of studies examining the impact of PPI use on the oral microbiota.

Study	Study Design	Patient Characteristic	Confounders	Sample	Sequencing Methods	Alpha Diversity	Beta Diversity	Taxonomic Changes
Tsuda et al. (2015) [21]	Cross-sectional study	Total patients (*n* = 45), including 12 functional dyspepsia and six GERD patients taking a PPI for more than 2 years, 27 of 45 healthy volunteers.	Matching for age	Saliva	Barcoded 454 pyrosequencing, 16S rDNA, V1-V2	OTU number (ns)	Unweighted UniFrac distance (sig.) PCoA analysis (ns)	ns
Rosen et al. (2015) [24]	Cross-sectional study	Children undergoing bronchoscopy and gastrointestinal endoscopy for chronic cough (*n* = 116), 59 were receiving a PPI dose within 24 h of endoscopy.	-	Oropharyngeal swabs	Illumina Miseq sequencing, 16S rDNA, -	Shannon index (ns)	-	Increased prevalence: Butyrivibrio Increased abundance: Allobaculum, Bifidobacterium, Cloacibacterium, Janthinobacterium, Ralstonia, Rhodobacter, Rhodoferax, Streptococcus, Yersinia, and Zoogloea.
Mishiro et al. (2018) [22]	Prospective self-controlled trial	Healthy adults (*n* = 10) before and after four weeks of 20 mg esomeprazole once daily.	-	Saliva, periodontal pocket fluid	Illumina Miseq sequencing, 16S rDNA, V3-V4	Chao1 (ns) Shannon index (sig. in saliva)	UniFrac distance (ns) PCoA analysis (sig. in saliva)	Increase: Fusobacterium and Leptotrichia in periodontal pocket fluid Decrease: Neisseria, Veillonella, and Haemophilus in saliva
Kawar et al. (2021) [23]	Cross-sectional study	Total patients (*n* = 128), including 20 patients with GERD who used PPIs, and 16 who had GERD but did not use medication, 102 negative control subjects.	Matching for age, periodontal status, and edentualism.	Saliva	Illumina Miseq sequencing, 16S rDNA, V1-V3	Chao1 (ns) Shannon index (ns)	PCoA analysis (ns)	Decrease: *Prevotella melaninogenica*, *Prevotella pallens*, *Solobacterium moorei* and Leptotrichia in the GERD patients not using PPIs compared to negative controls.

Abbreviations: GERD, gastroesophageal reflux disease; PPI, proton pump inhibitor; OTU, operational taxonomic units; ns, not significant; sig., significant; PCoA, principal coordinate analysis. -, not reported.

### 2.2. PPIs and Gut Microbiota

In contrast to the few studies with small sample sizes on oral microbiota, the majority of these studies addressed the influence of PPIs on the gut microbiota and were performed on fecal samples. Two large population-based cohort studies have yielded compelling evidence supporting the notion that PPI use can significantly alter the composition of the gut microbiome [11,12]. Imhann et al. analyzed the gut microbiota composition of 1815 adult individuals across three independent Dutch cohorts and found that PPI use is associated with a significant decrease in observed species and Shannon diversity. Taxonomic analysis showed that PPI use was related to statistically significant increases in several taxa, including the Actinomycetales order, the Streptococcoceae and Micrococcoceae families, the Rothia genus, and *Lactobacillus salivarius*. Furthermore, these microbial alterations in the gut associated with PPI use remained statistically significant after adjusting for antibiotics and other commonly used drugs [11]. In another large-scale study, Jackson et al. investigated the relationship between PPI use and the gut microbiota by analyzing 16S rRNA in fecal samples from more than 1800 twin volunteers. After adjustment for family and twin structure, BMI, age, frailty, and gastrointestinal indications, the researchers found that PPI use (229 individuals) did not significantly impact the overall gut microbial diversity. However, at the taxonomic level, PPI use increased the abundance of the Streptococcaceae and Micrococcaceae families, and decreased the abundance of the Erysipelotrichaceae, Lachnospiraceae, and Ruminococcaceae families [12]. Notably, this study by Jackson et al. is the first to consider both host genetics and shared environmental effects when evaluating the impact of PPIs on gut microbiota [12]. Several small-scale observational studies have also demonstrated an association between PPI usage and composition alterations in the gut microbiota [21,25,26,27]. Zhang et al. pooled data from four studies that utilized 16S rRNA gene amplicon sequencing, and revealed common features of the PPI-specific microbiota, including negative associations with the Shannon diversity index and the abundance of bacteria from the Ruminococcaceae and Lachnospiraceae families [28].

The experimental design and longitudinal investigation of the same patients before and after PPI use may help to understand the causal relationship between PPIs and the gut microbiota. Freedberg et al. conducted a study in 12 healthy individuals who received omeprazole (40 mg twice daily) for four weeks and were then randomized to continue or discontinue PPIs for another four weeks. This study found no significant alterations in gut microbial species richness or community diversity but notable compositional changes. Specifically, both the four-week and eight-week PPI treatment groups exhibited significant increases in Enterococcaceae, Streptococcaceae, Micrococcaceae, and Staphylococcaceae. Conversely, a decrease in the Clostridiaceae family was consistently found in both treatment durations [29]. In a cohort study of 24 healthy individuals who received a 14-day course of omeprazole 20 mg daily, Reveles et al. found no effect on fecal microbial alpha diversity but observed changes in beta diversity and microbial composition; omeprazole significantly decreased the abundance of the families Bifidobacteriaceae, Erysipelotrichaceae, and Lachnospiraceae, and increased the abundance of Streptococcaceae [30]. Mishiro et al. also observed an increase in the abundance of fecal Streptococcus levels after four-week administration of esomeprazole [22]. Koo et al. investigated the impact of PPI use on the gut microbiome in a healthy, multi-ethnic Asian cohort of Chinese (*n* = 12), Malay (*n* = 12), and Indian (*n* = 10) ancestry. After seven-day administration of 20 mg omeprazole daily, there were increases in the class Bacilli, the order Lactobacillales, the family Streptococcaceae, the genera Streptococcus and Veillonella, and *Streptococcus vestibularis* and *Veillonella dispar*, which returned to baseline after omeprazole was stopped for another seven days [31]. Furthermore, Shi et al. investigated the influence of PPIs on the fecal microbiota in GERD by using internal transcribed spacer 1 sequencing (*n* = 65). Linear discriminant analysis effect size analysis revealed that Candida at the genus level was a biomarker for GERD patients who used PPIs [32].

To date, current evidence from both large observational and interventional studies indicates that PPIs alter the gut microbiota, especially increasing the abundance of multiple oral-originated microbiota taxa.

## 3. Influence of Oral–Gut Translocation on PPI-Induced Gut Microbiota Alteration

Increasing amounts of data have shown that the increased taxa after PPI use are more likely to be oral bacteria, not gut commensals. By analyzing 116 oral microbial samples, Jackson et al. demonstrated a significant shift in the composition of the fecal microbiota of PPI users to the oral microbiome [12]. Imhann et al. equally observed that the most striking change in PPI users was an increase in the order Lactobacillales, especially the family Streptococcaceae, which are typically abundant in the oral cavity [11]. It has been proposed that the increased presence of oral bacteria in the gut may be a direct consequence of PPIs’ impact on stomach acid [11]. In detail, the acidic environment of the stomach acts as a primary defense against the influx of exogenous microbes that occur during the consumption of food and oral mucus. By reducing stomach acidity, PPIs enable more bacteria to survive the disruption of this gastric acid barrier [11].

To assess the hypothesis that PPI use could influence the abundance of gut species by facilitating the translocation of oral microbiota. We performed a prospective interventional study in healthy adult volunteers [17]. In our study, a seven-day course of esomeprazole (40 mg once daily, *n* = 8) did not alter the alpha or beta diversity of gut microbiota. However, taxonomic analysis revealed that PPI administration led to an increased abundance of Streptococcus in the gut microbiota, with the increased Streptococcus species originating from the oral site or oral/nasal sites. Furthermore, microbial source tracking demonstrated that PPI use significantly enhanced the contribution of oral bacteria to the gut microbiota. When esomeprazole was administered along with mouthwash, the impact of oral bacteria on the gut microbiota was weakened. *Streptococcus anginosus* was identified as a significantly altered oral species. Using a mouse model, we demonstrated that PPI usage increased the abundance of *Streptococcus anginosus* in fecal and intestinal tissue samples, which correlated with significantly elevated intragastric pH and serum gastrin concentration [17]. These findings jointly support the hypothesis that PPIs alter the gut microbiota by disrupting the gastric acid barrier and promoting the translocation of oral bacteria to the gut.

Meanwhile, Zhu et al. compared the effects of omeprazole (20 mg daily, *n* = 23) and famotidine (20 mg daily, *n* = 26) through a longitudinal study; the latter is a histamine-2 receptor antagonist (H2RA) with less gastric acid suppression than PPIs. The authors revealed that PPI usage resulted in a significantly higher degree of oral-to-gut translocation, fostered the growth of specific oral species in the gut, and disturbed the gut microbiota to a greater extent than H2RA [33]. In another study, Otsuka et al. compared the effects of lansoprazole (30 mg once daily, *n* = 11) with the more potent potassium-competitive acid blocker vonoprazan (*n* = 9) on the gut microbiota of healthy adults. Compared to the baseline, there was an over 20-fold increase in the abundance of the Streptococcus genus after vonoprazan treatment as compared to an increase of approximately 5-fold after lansoprazole treatment. It is evident that vonoprazan exerts a stronger effect in promoting the translocation of oral microbiota to the gut than lansoprazole [34]. Taken together, these data support the notion that gastric acid suppression facilitates the translocation of oral bacteria and disrupts the gut microbiota.

Apart from the indirect impact mediated by gastric acid suppression, it has been proposed that PPIs directly impact gut microbial composition by inhibiting specific commensal gut bacteria. In an in vivo study, Maier et al. demonstrated that PPIs could directly suppress the growth of taxa that were found to be reduced in PPI users but had a lesser effect on the taxa enriched in PPI users [35]. However, this direct inhibitory effect may not explain why PPI use is associated with an increase in various taxa originating from the oral cavity [11,12]. Moreover, elderly people with impaired or less functional gastrointestinal barriers exhibited a higher prevalence of oral bacteria, including Porphyromonas, Fusobacterium, and Pseudoramibacter, in their guts compared to healthy adults. This finding further suggests that impairment of the oral–gut barrier allows the translocation of oral microbiota to the gut [36].

Based on the available evidence, PPI use is associated with alterations in both the oral and gut microbiota (Table 2). In the majority of studies, these microbial changes were linked to altered abundances of specific taxa; however, there were conflicting findings regarding changes in microbial diversity [11,12,17,33,34]. Studies of the oral microbiota are limited by observational designs and small sample sizes, compared to the extensive consideration given to the influence on gut microbiota. In fecal samples, PPI-induced alterations in microbiota profiles have been well characterized in both interventional studies and large observational cohorts. The use of PPIs is associated with an increased abundance of multiple taxa originating from the oral cavity, including the genus Streptococcus [11,12,17,33,34]. Mechanistically speaking, oral bacterial translocation promoted by gastric acid suppression might be the primary reason for PPI-induced gut microbial alterations [17,33].

## 4. Oral–Gut Microbiota and PPI-Related Digestive System Complications

**Figure 1:**
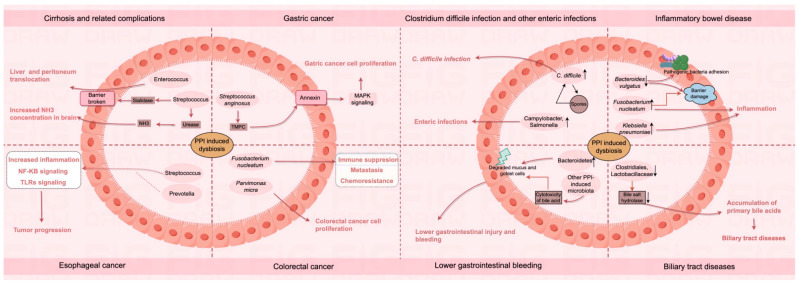
Possible roles of altered oral–gut microbiota in various PPI-related digestive system complications.

Gut microbiota play a crucial role in human health and disease [37]. As oral microbiota translocation induced by PPIs affects the gut microbiota [11,12,17], we investigated the potential role of oral microbiota in PPI-related digestive diseases (Figure 1).

### 4.1. Cirrhosis and Related Complications

Over one-third of patients with cirrhosis received acid suppression therapy [38,39]. Recently, inappropriate and prolonged administration of PPIs has been found to increase the risks related to cirrhosis.

Spontaneous bacterial peritonitis (SBP) is a serious complication of decompensated cirrhosis, characterized by an infection of the ascites without any intra-abdominal surgically treatable source [40]. Due to the lack of randomized controlled trials, Wong et al. recently conducted a meta-analysis focusing on longitudinal studies. A pooled analysis of five longitudinal studies demonstrated that the use of PPIs was associated with a 1.75-fold increased risk of SBP, and provided strong evidence for causal links based on the Bradford Hill criteria [39]. Moreover, Janka et al. utilized a long follow-up period (median 1155 days) and reported that the median time from the initiation of PPI therapy to the first episode of SBP was approximately one year [41]. Therefore, PPI use may elevate the risk of developing SBP in patients with cirrhosis.

Hepatic encephalopathy (HE) is a neuropsychiatric disorder that develops in patients with decompensated cirrhosis, and its severity ranges from minimal to overt. The first retrospective study by Dam et al. reported that the hazard ratio for developing HE in current PPI users versus non-users was 1.36 after adjusting for confounding factors [42]. Tsai et al. conducted a matched case-control study and discovered that PPI use was associated with an increased risk of developing HE in a dose-dependent manner [43]. A post hoc analysis of the ATTIRE trial revealed that patients who were using PPIs at baseline had a significantly higher incidence of developing Grade III/IV HE during hospital stays [44]. However, the authors admitted that the limitations of observational epidemiology could not be ignored even if they tried to investigate potential confounders [44]. The association between PPI use and the risk of developing HE requires further confirmation through well-designed, multicenter prospective studies.

It is speculated that PPIs also increase the mortality of cirrhosis. A meta-analysis including 28 studies with 260,854 cirrhotic patients demonstrated that PPI use was significantly associated with an increased risk of long-term mortality in these patients, but not associated with short-term mortality [39]. However, information on cause-specific mortality was not available, and future studies are needed.

There are conflicting results regarding PPI use and hepatocellular carcinoma (HCC) in cirrhosis. Shao et al. from Taiwan reported that the use of PPIs in patients who were not infected with hepatitis B or C increased the risk of HCC in a dose–response manner [45]. Li et al. from the United States reported that PPI use increased the risk of HCC in patients with chronic hepatitis C infections [46]. However, two studies from Taiwan, one from the United Kingdom, and one from Korea found no significant association between PPI use and HCC [47,48,49,50]. Conflicting results from observational studies on the association between PPI use and HCC may stem from differences in study design, inclusion and exclusion criteria, and confounding factors. To establish a clearer understanding of this relationship, more prospective, large-scale trials with long-term follow-up studies are warranted.

Both HE and SBP are associated with changes in the gut microbiota, which led to the hypothesis that gut microbiota alterations induced by PPI use may be responsible for the increased risk of cirrhosis related to PPI use. Bajaj et al. reported in detail on the impact of PPI use on the gut microbiota in liver cirrhosis using different study designs. The cross-sectional microbiota analysis included 137 cirrhotic patients (59 on PPIs) and 45 controls (17 on PPIs), indicating that, regardless of cirrhosis, PPI users exhibited a higher abundance of Streptococcaceae. According to the longitudinal microbiota analysis, initiation and use of omeprazole for more than two weeks in fifteen cases of decompensated cirrhosis increased the abundance of Streptococcaceae and Porphyromonadaceae compared to baseline levels. Conversely, discontinuing PPIs for two weeks significantly decreased the abundance of these oral-origin taxa [51]. Similarly, Yamamoto et al. showed that the use of PPIs increased oral-origin microbiota (Lactobacillus and Streptococcus) and decreased autochthonous microbiota (Dorea, Anaerotruncus, and Ruminococcus) [52].

Some members of the genus Streptococcus are important regulators of the gut–liver–brain axis in cirrhosis, as they can express urease, which is important for ammonia production and the pathogenesis of HE [53]. A previous study revealed that the abundance of *Streptococcus salivarius* was considerably greater in the gut microbiome of cirrhotic patients with minimal HE compared to those without HE, and revealed a positive correlation between the change in the quantity of this bacteria and the accumulation of ammonia [54]. Many orally originating bacteria, including *Veillonella atypica*, *Veillonella parvula*, and *Streptococcus* spp., possess an abundance of sialidase enzymes that break down O-glycans present on human mucins, potentially causing damage to the intestinal barrier. In a recent double-blind, randomized, placebo-controlled trial, rifaximin-α led to the regression of both overt and covert HE in patients with cirrhosis [55]. Mechanistically speaking, rifaximin-α inhibited the growth of these orally derived species in feces, including *Streptococcus* spp., *Veillonella atypica* and *parvula*, Akkermansia, and Hungatella, which are known to degrade due to their sialidase activity and have been associated with gut barrier damage and systemic inflammatory milieu [55]. On the other hand, the families Ruminococcaceae (genera Anaerotruncus and Ruminococcus) and Lachnospiraceae (genus Dorea) were associated with carbohydrate fermentation to produce short-chain fatty acids (SCFAs), which play important roles in maintaining intestinal barrier function, immune regulation, and anti-inflammatory effects [56]. In cirrhosis, as the severity of liver disease worsened, the abundance of these beneficial microbial taxa decreased and the associated decrease in SCFAs became more pronounced [57]. Llorente et al. provided additional data in mouse models, showing that PPIs promoted the progression of alcoholic liver disease and nonalcoholic fatty liver disease by inducing excess growth of intestinal Enterococcus. Mechanistically, an increasing number of Enterococcus translocated to the liver through the portal vein and bound to the pathogen recognition receptors on hepatic Kupffer cells, leading to interleukin (IL)-1β secretion and inflammation [58]. Based on these data, it is expected that PPIs increase the abundance of harmful oral-originated taxa that could outcompete beneficial microbial taxa in the gut and in turn contribute to the progression of cirrhosis. To date, causal connections between PPIs and oral–gut microbiota and cirrhosis-related complications remain largely hypothetical and require further investigation in larger cohorts and well-designed experimental studies.

### 4.2. Gastric Cancer

The impact of long-term PPI use on the development of gastric cancer (GC) has been extensively investigated [59,60]. Peng et al. performed a meta-analysis that included the largest number of studies, in which 16 studies (eight cohort and eight case-control studies) reported a 1.75-fold increased risk of GC in PPI users [61]. Pan et al. similarly demonstrated a 1.67-fold increase in GC risk through a meta-analysis of 15 observational studies [60]. In addition, the authors identified one interventional study by Moayyedi et al., in which pantoprazole use among patients taking aspirin or rivaroxaban did not increase the risk of GC during a median follow-up of 3.01 years [60,62]. Notably, this interventional trial was based on a group of participants receiving aspirin treatment, which may limit the generalizability of the results [62]. Overall, the available evidence is supportive of a small significantly increased risk of GC with PPI use, mostly from observational studies.

One hypothesis regarding the link between long-term use of PPIs and the development of GC is that reduced gastric acid secretion leads to secondary hypergastrinemia which has a direct nutritional effect on enterochromaffin-like (ECL) cells. Zheng et al. reported on 27,283 participants from 10 studies and showed that maintenance therapy with PPIs for more than six months was associated with a threefold increased risk of ECL cell hyperplasia [63]. There seems to be a PPI-gastrin-ECL cell hyperplasia pathway that simulates gastric neuroendocrine tumors [64]. Alternatively, perturbations in the gastrointestinal microbiota caused by long-term PPI use may facilitate the development of cancer. Chen et al. and Zhou et al. reviewed the scientific evidence for the roles of the microbiome in gastric carcinogenesis and found that the genera Streptococcus, Lactobacillus, Prevotella, and Veillonella were more prominent in GC patients both in fecal and gastric samples [65,66]. In particular, the abundances of the genera Streptococcus and Lactobacillus, which originate in the oral cavity, significantly increased in PPI users. Rosen et al. and Sterbini et al. indicated that the genus Streptococcus was significantly enriched in both PPI-treated gastric biopsies and gastric fluid samples, and this increase was independent of *Helicobacter pylori* [24,67]. Recently, Fu et al. reported for the first time that infection with oral *S. anginosus* could also promote gastric inflammation and tumorigenesis through mitogen-activated protein kinase signaling [68]. This finding implies that these translocated oral microorganisms may promote gastric carcinogenesis in the presence of PPI use. However, the mechanism of PPI-related microbial changes in GC development has not been extensively studied.

To date, observational epidemiology has limitations in explaining causation, and evidence from interventional studies is lacking. It is still unclear whether PPI-induced changes in the microbiota represent a risk factor for GC or its consequences, and warrants further experimental investigation.

### 4.3. Esophageal Cancer

Earlier observational studies and meta-analyses, primarily conducted in Western countries, seemed to deny the carcinogenic effect of PPIs on esophageal cancer [69]. A meta-analysis encompassing eight cohort studies demonstrated that PPIs can significantly decrease the risk of high-grade dysplasia and/or esophageal adenocarcinoma in patients with Barrett’s esophagus (BE) by nearly half [69]. Notably, original studies from Asia were not included in the meta-analyses. In Asia, the predominant type of esophageal cancer is squamous cell carcinoma, unlike in Western countries. In an Asian population, a previous case-control study in Taiwan revealed that PPI users exhibited a 3.83-fold greater risk of developing esophageal cancer than non-users [70]. A recently published case-control study from Korea, consisting of 811 esophageal cancer patients and 3244 controls, indicated that PPI users had 13.23-fold greater odds of esophageal cancers compared to nonusers. Subgroup analyses advocated that patients who did not experience gastroesophageal reflux were more likely to be associated with esophageal cancer due to the use of PPIs, suggesting that the carcinogenic effect of PPI use is more likely to be related to squamous cell carcinoma [71]. To date, current studies have shown a protective effect of PPIs on esophageal adenocarcinoma in the West and a carcinogenic effect on esophageal squamous carcinoma in Asia. Further evidence from prospective and interventional studies is needed to examine the specific role of PPIs in different types of esophageal cancer.

The mechanistic basis for the conflicting effects of PPIs on esophageal cancer is complicated. First, PPIs can theoretically alleviate the symptoms of gastroesophageal reflux and facilitate the healing of esophagitis by decreasing acid production, thus reducing the risk of developing metaplasia-dysplasia-adenocarcinoma in BE patients [69]. Second, PPI use has been shown to alter the esophageal microbiota, which may play a role in the development of cancer [72]. PPI use was associated with significant increases in the abundance of the Clostridiaceae, Lachnospiraceae, Microccocaceae, and Actinomycetaceae families and a decrease in the Comamonadaceae family in esophageal biopsies [73]. Microbiota were analyzed in 17 esophageal biopsy samples collected from 10 PPI users and seven non-users, and researchers found that *Prevotella* sp. and *Streptococcus* sp. were abundant in the PPI users [74]. There is a strong consensus in the literature that not only the esophageal microbiota, but also the microbiomes of other parts of the human body may be involved in the development of esophageal cancer. The review by Muszyński et al. provided insights into the search for the significant alterations in the oral and gut microbiome that may influence the occurrence of esophageal cancer [72]. At the species level, *Aggregatibacter actinomycetemcomitans*, *Streptococcus anginosus*, *Tannerella forsythia*, *and Treponema denticola* in the oral samples, as well as *Bacteroides fragilis, Escherichia coli, Akkermansia muciniphila, Clostridium hathewayi, Alistipes finegoldii*, *Prevotella intermedia*, and *Prevotella melaninogenica* were identified as microbial biomarkers for esophageal cancer [65,72,75]. Considering the altered oral microbiota between PPI-associated and esophageal cancer-associated microbial changes, *Streptococcus* sp. and *Prevotella* sp. may play a role in facilitating carcinogenesis in esophageal cancer through chronic inflammatory pathways [76]. Overall, the specific mechanisms responsible for esophageal cancer development in the abovementioned microbes are poorly described. Whether the oral-to-gut translocation of a specific taxon induced by PPI use contributes to protective or harmful effect on esophageal cancer is unclear and should be carefully validated in the future.

### 4.4. Colorectal Cancer

Thirteen observational epidemiological studies and associated meta-analyses have shown inconsistent findings regarding whether PPI use increases the risk of CRC [59]. The most recent meta-analysis by Guo et al. included 12 studies (four cohort and eight case-control studies) comprising 3,635,022 participants and showed that PPI use increased the risk of CRC 1.22-fold [77]. Recently, one study by Kwon et al. utilizing Korean national databases, which included 9374 CRC patients and 37,496 matched controls, revealed that PPI use was associated with a more than fivefold increase in the risk of developing CRC [78]. This finding is consistent with those of three previous studies conducted in East Asian populations [79,80,81]. However, Lee et al. and Babic et al. reported that PPI use did not increase the risk of CRC in the United Kingdom or the United States, respectively [9,82]. Further investigations are needed to present compelling evidence for such a causative relationship. In addition, Lin et al. performed a quantitative analysis and demonstrated that the concurrent use of PPIs contributed to a marginally significant 18% increase in all-cause mortality in CRC patients receiving fluorouracil-based regimens; conversely, PPI use had little influence on the survival of patients receiving capecitabine-based therapy [83]. One possible explanation suggests that concomitant PPIs may reduce the efficacy of specific chemotherapies, thus impacting CRC prognosis.

Regarding the risk of CRC, the elevated levels of gastrin and changes in the gut microbiota induced by PPIs may contribute to the development of CRC. Theoretically, gastrin exerts proliferative and antiapoptotic effects on various cancer cell types [84,85,86]. Nevertheless, some animal studies have shown that PPI-induced secondary hypergastrinemia does not seem to promote the growth and metastasis of CRC [84,87]. Recent evidence has confirmed the presence of various oral microbes in the development of CRC [88,89,90]. At the genus level, fecal samples and cancer tissues from CRC patients were enriched in Fusobacterium, Parvimonas, Porphyromonas, Streptococcus, and Peptostreptococcus. In addition, cancer tissues were also enriched in Gemella and Granulicatella. At the species level, *Fusobacterium nucleatum (F. nucleatum)*, *Parvimonas micra (P. micra)*, *Porphyromonas asaccharolytica*, *Porphyromonas gingivalis*, *Prevotella intermedia*, *Peptostreptococcus anaerobius*, *Peptostreptococcus stomatis*, *Streptococcus sanguinis, Solobacterium moorei*, *Gemella morbillorum*, *and Actinomyces odontolyticus* were enriched in CRC patients, and most of them have been proven to play important roles in promoting tumor transformation and progression [90]. When the abovementioned carcinogenic species were compared with the PPI-associated oral–gut translocated microbial taxa reviewed here, *F. nucleatum* and *P. micra* were found to be translocated from the oral cavity to the gut, promoted by PPI usage [32]. Wang et al. recently summarized that *F. nucleatum,* a common oral bacterium, could contribute to tumor proliferation and metabolism, remodel the immune microenvironment, and facilitate metastasis and chemoresistance in the tumorigenesis and development of CRC [91]. Furthermore, Zepeda-Rivera et al. reported that tumor-isolated strains predominantly belong to the *F. nucleatum subspecies animalis* (Fna), and specifically Fna C2, which has an oncogenic function [92]. Chang et al. demonstrated that *P. micra* could facilitate the development of CRC through the miR-218-5p/Ras/ERK/c-Fos pathway [93]. Bergsten et al. found in vitro and in vivo that *P. micra* affected the DNA methylation profile of promoters of key oncogenes, tumor-suppressor genes, and genes involved in epithelial–mesenchymal transition [94]. Future studies using murine models of CRC will delineate whether *F. nucleatum* and *P. micra* mediate the increased CRC risk in PPI users. With regard to the effects of PPIs on the prognosis of CRC, Kichenadasse et al. speculated that PPIs may reduce the intratumoral concentration of cytotoxic drugs by inhibiting uptake transporters and altering the pH of the tumor microenvironment [95]. This hypothesis requires further testing in preclinical studies.

### 4.5. Clostridium Difficile Infection and Other Enteric Infections

*Clostridium difficile* (*C. difficile*), a gram-positive anaerobic bacterium, is a common cause of nosocomial infection. Published epidemiological studies have indicated that PPI treatment is positively associated with CDI occurrence [96,97]. A meta-analysis including 67 studies indicated a 2.34-fold increase in the risk of CDI and a 1.73-fold increase in the risk of recurrent CDI in PPI users [98]. Moreover, Inghammar et al. showed that the increased risk of CDI was still significant one year after PPI treatment ended [98]. Recently, Huang et al. showed that the duration from PPI use to CDI occurrence ranged from 14 to 15 days, emphasizing the need to be vigilant about the risk of CDI in patients who take PPIs for more than two weeks [96]. The connection between PPI usage and CDI seems evident. Notably, the US Food and Drug Administration has cautioned that PPI therapy may be tied to a heightened risk of diarrhea associated with *C. difficile* [99].

The relationship between the use of PPIs and community-acquired enteric infections has also been discussed. A pooled analysis of nine observational studies showed that PPI users have a 4.28-fold increased risk of developing community-acquired enteric infections, especially *Salmonella* infection and *Campylobacter* infection [100]. Moreover, a meta-analysis of 12 studies involving 22,305 participants demonstrated that PPI use increased the risk of intestinal infection by approximately 74% for multidrug-resistant microorganisms, including Enterobacterales and vancomycin-resistant Enterococci [101].

A possible mechanism is proposed as follows. First, PPIs elevate the pH by reducing gastric acid secretion, diminishing the bactericidal effect of the gastric acid and enabling exogenous bacteria to enter the gastrointestinal tract more easily. According to in vitro tests, *C. difficile* spores cannot sporulate at a low gastric pH, while a higher gastric pH favors sporulation and germination of vegetative forms of the bacterium. Likewise, *Campylobacter* and *Salmonella* exhibit better survival rates at a higher pH [100,102]. Additionally, PPI use disrupts the equilibrium of the normal gut microbiota, diminishing the antagonistic effect against exogenous bacteria. The effectiveness of fecal transplantation in treating CDI confirms the importance of a healthy gut microbiota in providing a protective environment against enteric infections [103]. In short, it is reasonable to be vigilant about the potential risk of enteric infection when prescribing PPIs.

### 4.6. Inflammatory Bowel Disease

IBD is a chronic inflammatory disease of the intestine, including ulcerative colitis (UC) and Crohn’s disease (CD) [104]. The etiology of IBD remains uncertain, possibly involving genetic and environmental factors, altered microbiota, and immune imbalances [104]. Recent large population-based studies have implicated PPIs as potential risk factors for IBD [105,106]. Xia et al. conducted a prospective cohort study encompassing more than 640,000 individuals with a median followed time of 12 years. The authors showed that compared to those who did not use PPIs, patients who used PPIs had a 42% increased risk of IBD, and compared to H2RAs patients, patients who used PPIs had a 38% increased risk of IBD [106]. This study has several strengths that could provide more robust evidence for its findings, such as comprehensive adjustment for possible confounders, the propensity score-matching method, and a two-year lagged analysis of the exposure. Schwartz et al. reported a similar increase in the risk of IBD in children who were prescribed PPIs [107]. Moreover, Onwuzo et al. showed that PPI users had a 2.02- and 2.79-fold increased risk of developing UC and CD, respectively [108]. Studies have also shown that PPI use may exacerbate disease severity when prescribed to adults with a history of IBD [109,110]. Juillerat et al. reported that patients with IBD taking a new prescription for PPIs showed a doubling in the risk of a severe flare than those who were not prescribed PPIs [109]. Lu et al. showed that IBD patients taking PPIs were less likely to experience remission and more likely to be hospitalized while on infliximab therapy [111].

One plausible hypothesis is that PPIs induce alterations in the gut microbiota, which may trigger an inflammatory and immune response contributing to the development of IBD. For the first time, Son et al. attempted to explain whether gastric acid suppressants regulate colitis mediated by altered gut microbiota in an animal study. In this study, rabeprazole significantly aggravated colitis in mouse models induced by dextran sulfate sodium (DSS) and dinitrobenzene sulfonic acid, and damaged intestinal epithelial barrier function. Mechanistically, rabeprazole decreased the relative abundance of *Bacteroides vulgatus* (*B. vulgatus*). Conversely, supplementation with *B. vulgatus* reduced intestinal inflammation by inhibiting the epithelial adhesion of pathogenic bacteria [112]. A recent study suggested that PPIs can affect the gut microbiota via the oral–gut axis by inhibiting gastric acid, thus disturbing the acid barrier [17]. Additionally, it has been reported that oral microbiota are enriched in the intestine, such as *F. nucleatum*, *Klebsiella pneumoniae*, *Haemophilus parainfluenzae*, which are potential common microbial characteristics for PPI users and IBD patients [33]. Some studies have demonstrated that *F. nucleatum* could activate the IL-17F/NF-κB pathway and M1 macrophage polarization, leading to disruption of the intestinal mucosal barrier and inflammatory cytokines release, thus aggravating DSS-induced colitis [113,114]. Kitamoto et al. reported that specific *Klebsiella pneumoniae* ectopically colonized the gut after migrating from the oral cavity, activating inflammasome-mediated inflammation [15]. Therefore, PPIs are expected to play a crucial role in IBD development by regulating the gut microbiota through the oral–gut axis, inducing intestinal immune response and exacerbating intestinal inflammation. More fundamental research is necessary to elucidate the role and pathogenic mechanisms of specific microbes in PPI-related risk of IBD.

### 4.7. Lower Gastrointestinal Bleeding

PPIs have been proven to be efficacious in treating and preventing upper gastrointestinal complications in high-risk patients receiving nonsteroidal anti-inflammatory drugs (NSAIDs), due to their potent and long-lasting inhibitory effect on gastric acid secretion. Currently, however, there are concerns about possible harmful effects on the lower gastrointestinal tract. Recently, we conducted a meta-analysis of 14 studies comprising 1996 subjects and found that concomitant use of PPIs with NSAIDs significantly increased the prevalence and number of small-bowel injuries detected by endoscopy, and decreased hemoglobin levels. However, our analysis did not establish a significant connection between PPI use and the risk of clinically significant small-bowel hemorrhages [115]. Jung et al. conducted another meta-analysis including 12 studies with 341,063 participants and found that PPI use was associated with an increased risk of lower gastrointestinal bleeding, particularly small bowel bleeding [116]. However, as the authors admitted, the definition of lower gastrointestinal bleeding differed between the studies. Four out of the 12 studies included in this meta-analysis defined small bleeding as erosion or ulcers detected by capsule endoscopy, which may potentially overestimate the true risk of small bowel bleeding related to PPIs. Further research is needed to determine the clinical significance of the effects of PPIs on lower gastrointestinal bleeding.

Existing studies have shown a strong connection between the gut microbiota and PPI-related lower gastrointestinal bleeding. Wallace et al. provided evidence that PPIs exacerbate the risk of small-bowel injury through the dysregulation of the gut microbiota [117]. Yoshihara et al. reported that the use of lansoprazole significantly increased the relative abundance of Bacteroidetes and resulted in a reduction in the thickness of the mucus layer and a decrease in the number of goblet cells, thereby promoting intestinal damage. Additionally, the administration of *Bifidobacterium bifidum* improved PPI-related intestinal injury [118]. Blackler et al. further demonstrated that PPI-induced microbiota alterations significantly promoted bile cytotoxicity and intestinal mucosal injury [119]. However, the majority of studies have been conducted using animal models, and further evidence with a high level of hierarchy is needed.

### 4.8. Biliary Tract Diseases

Several epidemiological studies have investigated the relationship between PPI use and biliary tract diseases, including cholelithiasis, biliary duct infection, and cancer. A large prospective cohort study involving 470,000 participants showed that after confounding adjustments, regular PPI use increased the risk of cholelithiasis, with an absolute risk of 3.39/1000 person-years [120]. A propensity score-matching study similarly reported that PPI use was closely related to a significantly higher risk of recurrence and a shorter recurrence-free period of common bile duct stones in patients treated with endoscopic sphincterotomy [121]. Given that cholelithiasis is responsible for the majority of cases of acute cholecystitis, PPIs may theoretically increase risk of biliary duct infection. Chuang et al. performed a case-control study using data from Taiwan’s national health insurance database. They discovered that PPI users had a 1.23-fold higher risk of developing cholecystitis compared to non-users after adjusting for comorbidities [122]. Min et al. utilizing Korean national databases involving 584,723 individuals and observed PPI use increased the risk of cholangitis approximately sixfold [123]. This result was augmented by those of Hakuta et al. and Sbeit et al. [124,125]. Hakuta et al. further indicated that the occurrence of cholangitis caused by multidrug-resistant bacteria was significantly higher in regular PPI users than in non-regular PPI users [124]. Additionally, correlations of PPIs and biliary duct cancer have also been reported, which may be related to PPI-induced cholelithiasis and biliary tract infections. One Swedish population-based cohort and two case-control studies in China indicated that PPI use was associated with a 1.5-fold higher likelihood of developing gallbladder cancer and cholangiocarcinoma compared to non-users [126,127,128]. Nevertheless, He et al. conducted a summary analysis of three prospective cohort studies, involving over 600,000 participants, and found that regular PPI use was not associated with risk of biliary tract cancer or any subtypes according to multivariate analysis or inverse probability of treatment weighting analysis [129]. Thus, there seems to be reason to doubt that previous findings of increased biliary duct cancer risk might be due to residual confounding or reverse causality. However, additional prospective studies with extended follow-up durations may be necessary to clarify these discrepancies.

The precise mechanism linking PPI use to biliary tract diseases has yet to be fully elucidated. One potential explanation is that PPI-induced alterations in the gut microbiota may subsequently contribute to an increased susceptibility to biliary tract diseases. Yang et al. indicated that gut microbial and metabolic changes resulting from long-term PPI exposure were similar to those resulting from a high-fat diet, which are identified risk factors for gallstone formation and cancer [130]. It has also been confirmed that long-term PPI exposure induced precancerous lesions of biliary duct in mice, including ductal epithelial proliferation, focal bile duct stenosis formation, and bile duct obstruction, which may further promote the formation of gallstones [130]. Additionally, the gut microbiota–bile acid axis may mediate the aforementioned risk association. He et al. found that PPI use reduced the abundances of some bacterial taxa known to alter bile salt hydrolase gene expression, such as Clostridiales and Lactobacillaceae, subsequently impairing bile acid metabolism and potentially leading to the accumulation of primary bile acids [131]. Therefore, PPI-related imbalances in the microbial environment and bile acids may jointly promote the development of biliary tract diseases.

Overall, the current literature primarily supports the associations between PPIs and several digestive diseases; however, most of the results were obtained from observational and retrospective studies that lacked sufficient information on confounding factors, or from large-scale epidemiological databases that were prone to residual confounding factors. Potential confounders include indications for using PPIs, overall health status, comorbidities, and other medications. More rigorous clinical studies and higher quality evidence are needed to establish the strength and cause-and-effect relationships of this correlation.

## 5. Conclusions and Future Directions

In this manuscript, we have reviewed current research on how PPIs shape the gut microbiota, with a specific focus on their impact on the oral–gut microbiota. An increasing number of studies have demonstrated that PPIs can alter the gut microbiota by promoting oral microbiota translocation. We also review the current evidence on PPI-induced oral–gut microbiota changes, which contribute to several digestive diseases including cirrhosis, gastrointestinal malignancies, IBD, enteric infections, lower gastrointestinal bleeding, and biliary tract diseases. Considering that some evidence is relatively weak, larger-scale, prospective, and well-designed trials are needed to understand the causal relationship between PPIs and digestive system diseases. On the other hand, the involvement and mechanism of the oral–gut microbiota in PPI-associated digestive diseases remain poorly described. Further in vivo and in vitro studies are required to identify the specific disease-associated strains or microbial metabolites of oral and gut microbiota in PPI-related digestive diseases. These findings could also serve as biomarkers for diagnosis and prevention. Additionally, further research evaluating the underlying mechanisms and molecular targets is expected to significantly contribute to the development of novel therapeutic strategies.

## Figures and Tables

**Table 2:** Summary evidence regarding oral–gut microbial translocation in the presence of PPIs.

Study	Study Design	Patient Characteristic	Sample	Methods	Translocated Oral Microbes in the Gut		Major Findings
					Genus	Species	
Imhann et al. (2016) [11]	Cross-sectional study	A total of 1815 individuals (211 PPI users) in three independent cohorts from the Netherlands: LifeLines-DEEP study, IBD cohort, IBS case-control study.	Oral cavity mucus samples and Feces	Illumina Miseq sequencing, 16S rDNA, V4	Rothia, Streptococcus, Actinomyces, Alloscardovia, Lactobacillus, Oribacterium, Granulicatella, Scardovia, Staphylococcus, Atopobium, Corynebacterium	*Rothia mucilaginosa,* *Rothia dentocariosa,* *Lactobacillus salivarius,* *Streptococcus sobrinus,* *Streptococcus anginosus, Staphylococcus aureus, Staphylococcus epidermidis, Lactobacillus reuteri, Lactobacillus vaginalis, Lactobacillus delbrueckii, Streptococcus infantis, Atopobium rimae, Staphylococcus haemolyticus.*	Multiple oral bacteria were over represented in the fecal microbiome of PPI users.
Jackson et al. (2016) [12]	Cross-sectional study	1827 healthy twins from the TwinsUK cohort.	Feces	Illumina Miseq sequencing, 16S rDNA, V4	Rothia, Streptococcus, Scardovia, Granulicatella, Oribacterium, Lactobacillus, Corynebacterium	*Rothia mucilaginosa,* *Streptococcus anginosus*	PPI use was associated with a significant increase in the abundance of oral and upper gastrointestinal tract commensals in gut commensals.
Otsuka et al. (2016) [34]	Prospective interventional trial	*Helicobacter pylori* IgG-negative healthy individuals (*n* = 20) taking four-week of 30 mg lansoprazole (*n* = 11) or 20 mg vonoprazan daily (*n* = 9).	Feces	Illumina Miseq sequencing, 16S rDNA, -	PPI group: Streptococcus, Carnobacterium, Oribacterium Vonoprazan group: Actinomyces, Rothia, Granulicatella, Streptococcus	*-*	Oral microbiome is more abundant in the gut microbiome after vonoprazan treatment as compared with lansoprazole treatment.
Xiao et al. (2024) [17]	Prospective self-controlled trial	Healthy adults (*n* = 16) taking 7-day course of 40 mg esomeprazole once daily.	Saliva and Feces	Illumina Miseq sequencing, 16S rDNA, V3-V4	Streptococcus, Gemella	*Streptococcus anginosus,* *Streptococcus parasanguinis clade 411,* *Streptococcus salivarius,* *Streptococcus vestibularis,* *Streptococcus mitis,* *Streptococcus sp. HMT 061,* *Streptococcus oralis subsp. dentisani clade 398,* *Streptococcus oralis subsp. dentisani clade 058,* *Lactococcus lactis*	PPI administration increased Streptococcus abundance in gut microbiota, and the increased species of Streptococcus were found to be from the oral site or oral/nasal sites, in which Streptococcus anginosus was identified as the significantly changed species.
Zhu et al. (2024) [33]	Prospective randomized controlled trial	Healthy adults (*n* = 49) before and after 7-day course of 20 mg omeprazole (*n* = 23) or 20 mg famotidine daily (*n* = 26).	Saliva and Feces	Shotgun metagenomic sequencing	PPI group: Streptococcus, Rothia H2RA group: Streptococcus	PPI group: *Actinomyces bouchesdurhonensis,* *Actinomyces oris,* *Actinomyces SGB17168,* *Actinomyces sp ICM58,* *Actinomyces sp S6 Spd3,* *Trueperella pyogenes,* *Rothia mucilaginosa,* *Isoptericola variabilis,* *Gemella sanguinis,* *Abiotrophia defective,* *Streptococcus constellatus,* *Streptococcus cristatus,* *Streptococcus mitis,* *Streptococcus sanguinis,* *Streptococcus sp 263 SSPC,* *Mogibacterium diversum,* *Solobacterium SGB6833,* *Megasphaera micronuciformis,* *Parvimonas micra,* *Fusobacterium nucleatum,* *Fusobacterium pseudoperiodonticum, Haemophilus sputorum* H2RA group: *Actinomyces,* *Bouchesdurhonensis,* *Rothia mucilaginosa,* *Isoptericola variabilis,* *Gemella sanguinis,* *Solobacterium SGB6833*	PPI usage led to a significantly higher extent of oral-to-gut transmission and promoted the growth of specific oral microbes in the gut than H2RA usage.

Abbreviations: GERD, gastroesophageal reflux disease; PPI, proton pump inhibitor; H2RA, histamine-2 receptor antagonist. -, not reported.
